# The Effect of Periodontitis on Fibroblast Growth Factor 23 Levels in Predialysis Chronic Kidney Disease Patients

**DOI:** 10.7759/cureus.65166

**Published:** 2024-07-23

**Authors:** Wan Asma Wan Abdul Azim, Nur Karyatee Kassim, Haslina Taib, Nurul Huda Abdullah, Nur Amirah Che Abdul Aziz, Hanim Afzan Ibrahim

**Affiliations:** 1 Department of Chemical Pathology, Universiti Sains Malaysia School of Medical Sciences, Kubang Kerian, MYS; 2 School of Dental Sciences, Universiti Sains Malaysia, Kubang Kerian, MYS; 3 Unit of Periodontics, School of Dental Sciences, Universiti Sains Malaysia, Kubang Kerian, MYS; 4 Department of Internal Medicine, Universiti Sains Malaysia School of Medical Sciences, Kubang Kerian, MYS; 5 Department of Dentistry, Ministry of Health Malaysia, Putrajaya, MYS

**Keywords:** predialysis, bone mineral markers, fibroblast growth factor 23, periodontitis, chronic kidney disease

## Abstract

Introduction

Chronic kidney disease (CKD) is known to cause an increase in fibroblast growth factor 23 (FGF23). Periodontitis, a condition recognized as a risk factor for CKD, is also potentially associated with the increment of FGF23. This study aims to compare FGF23 levels in CKD patients with and without periodontitis and non-CKD patients with and without periodontitis. Correlation with serum phosphate, calcium, and intact parathyroid hormone (iPTH) was assessed. Additionally, associations between FGF23, calcium, phosphate, iPTH, creatinine, urea, plaque score, and bleeding score with periodontitis in CKD patients were determined.

Method

A total of 124 participants were categorized into four groups: CKD patients with periodontitis (n=31), CKD patients without periodontitis (n=32), periodontitis patients without CKD (n=32), and healthy population (n=29). The selected CKD patients include those from stages 3 and 4 (predialysis) patients. Serum levels of FGF23, calcium, phosphate, iPTH, creatinine, and urea were analyzed. Oral examinations were conducted to determine the presence and absence of periodontitis and assess plaque and bleeding scores.

Result

A significantly higher level of FGF23 was found in CKD compared to non-CKD groups; however, no difference was observed with the presence of periodontitis in both CKD and non-CKD. There was no significant correlation found between FGF23 and serum calcium, phosphate, or iPTH concerning periodontal status. Apart from the bleeding score, there was no association between FGF23, calcium, phosphate, iPTH, creatinine, urea, and plaque score with the presence of periodontitis in CKD patients.

Conclusion

The presence of periodontitis was not associated with higher FGF23 levels in CKD patients. Changes in FGF23, calcium, phosphate, iPTH, creatinine, urea, and plaque score could not be attributed to the presence of periodontitis in CKD patients.

## Introduction

Fibroblast growth factor 23 (FGF23), a phosphatonin, is a bone-derived endocrine hormone crucial for circulatory phosphate and calcium homeostasis. It reduces renal phosphate reabsorption and inhibits 1α-hydroxylase activity, leading to suppressed 1,25(OH)2D (calcitriol) formation, which is the active form of vitamin D. FGF23 is markedly greater in chronic kidney disease (CKD) patients than in healthy populations and begin to rise in the early stages of CKD [1,2]. Aside from its role in mineral homeostasis maintenance, FGF23 contributes to chronic inflammation and is linked to an increased risk of all-cause mortality and cardiovascular events in CKD patients [3].

Periodontitis on the other hand is a common infectious condition that causes gingival inflammation as well as loss of supportive connective tissues such as the periodontal ligament and alveolar bone. This disease is linked to systemic inflammation, which can exacerbate renal dysfunction and is recognized as a modifiable risk factor for CKD [4]. Emerging studies suggest a relationship between periodontitis and elevated FGF23 levels [5,6]. However, the specific contribution of periodontitis to increased FGF23 levels in CKD patients remains unclear due to limited research on this subject.

CKD patients often suffer from disturbances in mineral metabolism, including elevated phosphate levels and secondary hyperparathyroidism. FGF23 plays a significant role in these processes by regulating phosphate and vitamin D metabolism. Understanding the factors that influence FGF23 levels could lead to better management of mineral dysregulation in CKD. Since both FGF23 and periodontitis are linked to inflammatory processes, investigating their interplay could uncover new pathways of inflammation-driven CKD progression. Periodontitis is a modifiable condition that can potentially be treated or prevented. If periodontitis is found to significantly influence FGF23 levels and subsequently CKD progression, it could present a novel therapeutic target for improving outcomes in CKD patients.

This study aims to compare the level of FGF23 in predialysis CKD patients with periodontitis, predialysis CKD patients without periodontitis, periodontitis patients without CKD, and healthy population and determine their correlation with phosphate, calcium, and intact parathyroid hormone (iPTH). We also aim to determine the association between FGF23, calcium, phosphate, iPTH, creatinine, urea, plaque score, and bleeding score with the presence of periodontitis in predialysis CKD patients.

## Materials and methods

This observational study was conducted at the medical specialist clinic and dental clinic at Hospital Universiti Sains Malaysia, Kelantan, Malaysia. The institutional ethical committee granted ethical approval for this study (JEPeM-USM code: USM/JEPeM/22060382), and written consent was obtained from each participant before enrolment. The study was conducted in accordance with the principles outlined in the Declaration of Helsinki, ensuring the ethical treatment of all participants.

An oral examination was conducted to confirm the presence and absence of periodontitis and determine plaque and bleeding scores based on the scoring of O’Leary et al. [7] and Ainamo et al. [8], respectively. In this study, predialysis CKD patients were classified as stages 3 and 4 CKD patients, diagnosed by medical physicians and not on regular dialysis treatment. Periodontitis is present when probing pocket depth is >3 mm, detectable at ≥2 teeth [9].

Inclusion criteria are males and females aged above 18 years old who are willing to participate and able to give consent. CKD patients receiving regular dialysis, patients in acute illness or acute kidney injury, post-parathyroidectomy, systemic diseases that acutely affect GFR (rapidly progressive glomerulonephritis, active glomerular disease, etc.), and/or oral health status (immunodeficiency syndrome and recurrent or active cancer), patients on medication that affect oral health status such as immunosuppressive drugs (corticosteroid drugs or chemotherapy), pregnant and lactating females, and those with uncontrolled diabetes (HbA1c > 8%) were excluded from this study.

Serum FGF23 was analyzed using the enzyme-linked immunosorbent assay (ELISA) method (ELK Human FGF-23 ELISA kit; ELK Biotechnology, Wuhan, China). Serum calcium, phosphate, urea, and creatinine were tested by spectrophotometric analysis (ARCHITECT C8000; Abbott Laboratories, Abbott Park, IL), whereas iPTH was assayed by electrochemiluminescence immunoassay (ECLIA) method (Cobas e-601; Roche Diagnostics, Basel, Basel-Stadt, Switzerland).

Overall statistical analysis was performed using SPSS (SPSS Inc., Chicago, IL, USA). The comparison of FGF23 was done using the Kruskal-Wallis test as the results were non-parametric. The Spearman test was used to determine the correlation between FGF23 and phosphate, calcium, and iPTH. Association between FGF23, calcium, phosphate, iPTH, creatinine, urea, plaque score, and bleeding score, and the presence of periodontitis in predialysis CKD patients was determined by logistic regression analysis.

## Results

A total of 124 participants were involved in this study with each falling into one of four groups: Group I, predialysis CKD patient with periodontitis (n=31); Group II, predialysis CKD patient without periodontitis (n=32); Group III, periodontitis patient without CKD (n=32); and Group IV, healthy population (n=29). The mean age of the participants was 50.5 (±15.8) years, with 61 males (49.2%) and 63 females (50.8%), and most of them were Malay (95.2%). Among CKD patients, stage 4 was the most frequent, with a creatinine mean of 222 (±61) µmol/L and urea of 10.6 (±3.9) mmol/L. The characteristic parameters according to the group are summarized in Table 1.

**Table 1:** Baseline characteristics of the participants SD: standard deviation, CKD: chronic kidney disease

	All groups (N=124)	Group I (n=31)	Group II (n=32)	Group III (n=32)	Group IV (n=29)
Age, years (mean±SD)	50.5 (15.8)	61.3 (9.9)	60.8 (10.5)	44.2 (13.8)	34.6 (10.5)
Gender (number (%))					
Male	61 (49.2)	24 (77.4)	19 (59.4)	13 (40.6)	5 (17.2)
Female	63 (50.8)	7 (22.6)	13 (40.6)	19 (59.4)	24 (82.8)
Race (number (%))					
Malay	118 (95.2)	28 (90.3)	30 (93.8)	32 (100)	29 (100)
Chinese	2 (1.6)	1 (3.2)	1 (3.1)	-	-
India	2 (1.6)	2 (6.5)	-	-	-
Others	2 (1.6)	-	1 (3.1)	-	-
iPTH, pmol/L (mean±SD)	-	6.94 (3.80)	6.60 (3.75)	3.60 (1.29)	3.36 (1.26)
Calcium, mmol/L (mean±SD)	-	2.27 (0.19)	2.27 (0.13)	2.26 (0.09)	2.29 (0.09)
Phosphate, mmol/L (mean±SD)	-	1.16 (0.18)	1.18 (0.18)	1.07 (0.16)	1.12 (0.17)
CKD stage (number (%))					
3	-	11 (35.5)	12 (37.5)	-	-
4	-	20 (64.5)	20 (62.5)	-	-
Creatinine, umol/L (mean±SD)	-	228 (63)	216 (59)	-	-
Urea, mmol/L (mean±SD)	-	10.6 (4.0)	10.3 (4.0)	-	-

The median FGF23 levels were highest in Group II at 124.22 pg/mL (interquartile range (IQR): 132.16), followed by Group I at 109.90 pg/mL (IQR: 293.16), Group IV at 39.20 pg/mL (IQR: 47.47), and Group III at 33.59 pg/mL (IQR: 47.27). Although there was a statistically significant difference in FGF23 levels among the four groups, post hoc analysis showed no significant difference in FGF23 levels related to the presence or absence of periodontitis in CKD and non-CKD groups. Table 2 shows the comparison of median serum FGF23 between the four groups.

**Table 2:** Comparing median serum FGF23 between the four groups FGF23: fibroblast growth factor 23, IQR: interquartile range

Group	Number	Median FGF23 (pg/mL) (IQR)	X2 statistics (df)*	p-value
Group I	31	109.90 (293.16)	42.689	<0.001
Group II	32	124.22 (132.16)		
Group III	32	33.59 (47.27)		
Group IV	29	39.20 (47.47)		

The Pearson correlation coefficient (r) for FGF23 and iPTH in the CKD group was 0.269 (p=0.034), indicating a weak positive correlation. In the non-CKD group, the correlation was not significant (r=0.120, p=0.358). No significant correlations were found between FGF23 and phosphate, calcium, and iPTH when analyzed separately in the four groups. Table 3 summarizes the correlation of serum FGF23 with phosphate, calcium, and iPTH in each group.

**Table 3:** Correlation between serum FGF23 and serum phosphate, calcium, and iPTH FGF23: fibroblast growth factor 23, iPTH: intact parathyroid hormone

Group	Phosphate		Calcium		iPTH	
	r	p	r	p	r	p
Group I	-0.124	0.506	-0.041	0.827	0.155	0.415
Group II	0.306	0.089	-0.099	0.591	0.250	0.167
Group III	0.127	0.487	0.007	0.968	0.254	0.160
Group IV	0.108	0.577	-0.133	0.493	0.001	0.996

Table 4 shows the result of the analysis in determining the association between FGF23, calcium, phosphate, iPTH, creatinine, urea, plaque score, and bleeding score, and periodontitis in predialysis CKD patients. Based on simple logistic regression analysis, three variables with a p-value of <0.25 were included in multiple logistic regression; they were FGF23, plaque score, and bleeding score. The analysis was then carried out using the forward likelihood-ratio model method, which showed that only the bleeding score was significant.

**Table 4:** Association between FGF23, calcium, phosphate, iPTH, creatinine, urea, plaque score, and bleeding score, and periodontitis in predialysis CKD patients by simple logistic regression FGF23: fibroblast growth factor 23, iPTH: intact parathyroid hormone, CKD: chronic kidney disease, SE: standard error, CI: confidence interval

Variable	Coefficient (B)	SE	Crude odd ratio (Exp B)	p-value	CI (95% for exp (B))	
					Lower	Upper
FGF23	0.002	0.002	1.002	0.243	0.999	1.005
Calcium	-0.276	2.948	0.925	0.657	0.002	244.98
Phosphate	0.414	1.924	1.513	0.830	0.035	65.643
iPTH	-0.91	0.095	0.337	0.337	0.759	1.099
Creatinine	0.005	0.006	1.005	0.419	0.993	1.017
Urea	0.045	0.102	1.046	0.657	0.857	1.278
Plaque score	0.023	0.015	1.024	0.117	0.994	1.054
Bleeding score	0.080	0.033	1.083	0.015	1.015	1.155

The Hosmer and Lemeshow goodness-of-fit test p-value was 0.141, indicating that the model was fit. The classification table showed that 68% of the models were correctly classified. The area under the receiver operating characteristic (ROC) curve was 0.75 (95% confidence interval (CI): 0.62-0.87), indicating that the model showed acceptable discriminative ability. Therefore, based on logistic regression analysis, the bleeding score was found to be associated with the presence of periodontitis with an increment of 1 bleeding score resulting in 1.08 times the odds of having periodontitis in predialysis CKD patients.

## Discussion

FGF23 level in predialysis CKD patients with periodontitis, predialysis CKD patients without periodontitis, and patients without CKD

In this present study, FGF23 levels were significantly higher in CKD patients compared to non-CKD subjects. This is consistent with many studies that showed that FGF23 had been associated with lower eGFR and is the earliest analyte affected in mineral metabolism in CKD prior to vitamin D, iPTH, calcium, and phosphate [2,10,11]. Elevated FGF23 in CKD is primarily driven by phosphate accumulation and FGF23 resistance, both consequences of renal impairment. The accumulation of phosphate occurs due to reduced phosphate excretion, as the kidney is the main organ responsible for phosphate hemostasis. This will signal osteocytes to produce more FGF23. FGF23 then binds to fibroblast growth factor receptor 1 (FGFR1), which is located at distal tubular cells and where its obligate co-factor, α-klotho, is present. This interaction will lead to the internalization of sodium phosphate co-transporters (NaPi2a and NaPi2c) from the proximal tubular cell's luminal plasma membrane, resulting in reduced phosphate reabsorption and, hence, phosphaturia [12,13]. FGF23 also inhibits the synthesis of 1,25-dihydroxyvitamin, resulting in reduced intestinal reabsorption of phosphate. The resistance to FGF23 observed in CKD is likely due to the decreased expression of its co-factor, α-klotho, which exacerbates the situation, leading to even higher levels of circulating FGF23. The decline in klotho level was postulated as a consequence of uremic toxicity and albuminuria tubule toxicity [14-17]. The unresponsive effect of FGF23 at the receptor level will cause osteocytes to release more FGF23, hence its accumulation in the bloodstream [18]. High FGF23 levels have adverse effects on cardiovascular health in CKD patients and are related to intermediate and severe complications, such as cardiac dysfunction, cardiovascular events, and mortality. The reason behind this association is the capability of FGF23 to induce inflammation, plus it is linked to vascular calcification and left ventricular hypertrophy in CKD patients [3].

We found no significant difference in the level of FGF23 in predialysis CKD patients with the presence or absence of periodontitis. A similar finding is also seen in the non-CKD group. The lack of significant difference in FGF23 levels in this study may suggest that the presence of CKD and its associated mechanisms of elevated FGF23 might overshadow the impact of periodontitis on FGF23 levels. Thus far, there is no comparable literature associating FGF23 in CKD patients with periodontitis. Ghalwash et al. [5] compared FGF23 in the non-CKD population and demonstrated a higher FGF23 level in the periodontitis group compared to the healthy population with a mean of 52.85 RU/mL versus 28.21 RU/mL, respectively, which decreased significantly following periodontal treatment. Another closely related study involving peri-implant disease revealed no significant difference in the level of FGF23 in peri-implant sulcus fluid in healthy subjects and those with peri-implantitis [19]. *Porphyromonas gingivalis *(*P. gingivalis*), a major pathogen in adult periodontitis, is thought to be the cause of high FGF23 in periodontitis. Kajiwara et al. [6] described in their study that the presence of *P. gingivalis* lipopolysaccharide (Pg-LPS) is related to the accumulation of FGF23 in the renal tubules and glomeruli, implying that Pg-LPS enhanced FGF23 accumulation in the renal tubule and thus increase its concentration in the circulation. This indicates a potential pathway through which periodontitis could influence FGF23 levels. However, the current study did not find a significant effect of periodontitis on FGF23 levels in CKD patients, which could be due to several factors, including the severity of periodontitis and the already elevated baseline levels of FGF23 due to CKD.

Treatment options for reducing FGF23 levels in CKD, such as phosphate binders, calcimimetics, and iron supplements, have been shown to be effective [20]. These treatments target the primary mechanisms of FGF23 elevation in CKD, addressing phosphate overload and modifying the effects of FGF23 resistance. The study suggests that while periodontitis treatment may not significantly impact FGF23 levels in CKD patients, the potential role of periodontal health in managing overall CKD progression and complications should not be overlooked. Periodontal disease is a source of chronic inflammation, which could contribute to the overall inflammatory burden in CKD patients. Therefore, maintaining good periodontal health could be beneficial as part of a comprehensive approach to managing CKD, even if it does not directly reduce FGF23 levels.

Future research should explore the impact of periodontitis severity on FGF23 levels and consider longitudinal studies to assess whether treating periodontitis in CKD patients has any long-term benefits on FGF23 levels and overall CKD outcomes. Additionally, investigating other inflammatory markers and pathways involved in periodontitis and CKD could provide a more comprehensive understanding of the interplay between these conditions.

Correlation of FGF23 with calcium, phosphate, and iPTH

The correlation between FGF23 and iPTH highlights the complex interplay of mineral metabolism in CKD. While FGF23 is known to play a role in regulating phosphate and vitamin D metabolism, iPTH is a key regulator of calcium and phosphate levels in the body. We found a fair positive correlation between FGF23 and iPTH in CKD patients (r=0.269, p=0.034), but no correlation in non-CKD individuals (r=0.120, p=0.358). This is consistent with the study done by Worung et al. [1], which revealed a low significantly positive correlation between FGF23 and iPTH in predialysis CKD (r=0.168, p=0.046), and a study by Hussain et al. [21], which also found a positive correlation between FGF23 and iPTH in hemodialysis patients (r=0.223, p=0.119). PTH and FGF23 may have opposing effects on one another. Apart from the kidney, FGF receptor and klotho are also expressed in the parathyroid gland, and the action of FGF23 on this receptor leads to a reduction level of PTH by decreasing PTH mRNA transcription [22]. However, in CKD, the downregulation of FGF receptors in the parathyroid glands limits this suppressive effect, resulting in elevated iPTH levels. Conversely, PTH can increase FGF23 production by stimulating osteocytes [23]. This reciprocal relationship explains the observed positive correlation between FGF23 and iPTH in CKD patients.

Our study did not find significant correlations between FGF23 and calcium or phosphate levels. This lack of correlation may be attributed to the early-stage compensatory mechanisms in CKD, where elevations in FGF23 and iPTH can maintain relatively normal serum calcium and phosphate levels [24]. In the predialysis stage of CKD, increased FGF23 promotes phosphate excretion and reduces 1,25-dihydroxyvitamin D levels, while elevated iPTH enhances calcium reabsorption and phosphate excretion. These compensatory actions can normalize serum calcium and phosphate, masking any direct correlations with FGF23 [25].

With regard to periodontal status, no significant correlations were found between FGF23 and calcium, phosphate, or iPTH in either CKD or non-CKD groups. This suggests that periodontitis does not significantly alter the interactions between FGF23 and these analytes. The inflammatory state induced by periodontitis may not be sufficient to disrupt the tightly regulated mechanisms governing mineral metabolism in CKD or non-CKD conditions. Additionally, the impact of periodontitis on systemic inflammation might not reach the threshold needed to affect FGF23 and its related pathways significantly.

While our findings indicate that periodontitis does not significantly influence the relationship between FGF23, calcium, phosphate, and iPTH, it remains crucial to consider the broader implications of chronic inflammation on CKD progression. Chronic inflammation, often exacerbated by periodontitis, can contribute to a pro-inflammatory milieu that affects overall health and disease progression. Future studies should delve deeper into the potential cumulative effects of chronic inflammatory conditions such as periodontitis on CKD outcomes, considering other markers of inflammation and mineral metabolism.

Association between FGF23, calcium, phosphate, iPTH, creatinine, urea, plaque score, and bleeding score, and periodontitis in predialysis CKD patients

Other than the bleeding score, there was no association found between FGF23, calcium, phosphate, iPTH, creatinine, and urea, and periodontitis in predialysis CKD using logistic regression. The lack of association between biochemical markers and periodontitis aligns with the results by Pradhan et al. [26] and Antonoglou et al. [27], which revealed non-significant results when comparing PTH in periodontitis and healthy groups. This suggests that traditional biochemical markers of CKD and mineral metabolism might not be sensitive indicators for periodontal disease in this population. The complex interactions of CKD-related metabolic changes might overshadow the impact of localized periodontal inflammation on systemic biochemical parameters.

Given their roles in mineral metabolism, it is plausible that the systemic alterations in FGF23 and iPTH in CKD patients are more reflective of the underlying renal pathology than of localized periodontal inflammation. During inflammation, the regulation of FGF23 involves a delicate balance between its synthesis and degradation. The mature FGF23 protein undergoes post-translational modifications that are crucial for maintaining this balance. One significant process in this regulation is the cleavage of FGF23 by proprotein convertases. These enzymes cleave mature FGF23 at a specific site between amino acids 179 and 180 [28].

CKD patients typically exhibit elevated FGF23 and iPTH levels as a compensatory mechanism to manage phosphate balance and calcium homeostasis. These systemic regulatory mechanisms might be dominant, making the additional influence of periodontitis negligible in comparison. As markers of kidney function, creatinine and urea reflect the glomerular filtration rate. Their levels are primarily influenced by renal function and protein metabolism rather than periodontal health. The absence of an association here indicates that periodontitis does not significantly alter renal function markers in predialysis CKD patients.

Similarly, the regulation of calcium and phosphate in CKD is tightly controlled by a network of hormones, including FGF23 and PTH. The influence of periodontitis on these serum levels is likely minimal compared to the profound alterations caused by declining renal function.

The significant association between bleeding score and periodontitis underscores the importance of clinical periodontal assessments. Bleeding on probing is a direct indicator of periodontal inflammation and an essential tool for diagnosing and monitoring periodontal disease. This finding emphasizes that despite the systemic complexities introduced by CKD, clinical signs of periodontal disease remain reliable indicators. Although not significantly associated with this study, plaque score is another critical clinical measure. Plaque accumulation is a precursor to gingival inflammation and periodontitis. The regular assessment of plaque score, alongside bleeding score, remains integral to periodontal disease management.

In conclusion, while biochemical markers such as FGF23, calcium, phosphate, iPTH, creatinine, and urea are essential for managing CKD, they do not predict periodontal disease presence. Clinical periodontal assessments, particularly bleeding and plaque scores, remain vital tools in the detection and management of periodontal disease in CKD patients. Integrating oral health into the comprehensive care plan for CKD patients is crucial for optimizing overall health outcomes.

The primary limitation of this study pertains to the relatively small sample size, which may reduce the generalizability of the findings. Additionally, the absence of graded assessments for periodontitis limits the ability to analyze the potential impacts of varying severity of periodontal disease on the outcomes. This study also did not include stage 5 CKD patients, where FGF23 levels are typically at their peak. Consequently, the results may not fully represent the entire CKD population, particularly those with more advanced diseases. Further investigation with enhanced methodological rigor may be requisite to validate the findings of this study in a more controlled research setting.

## Conclusions

In summary, this study found that elevated FGF23 levels in CKD patients are not associated with their periodontal condition. Moreover, the presence of periodontitis does not influence the relationship between calcium, phosphate, iPTH, and FGF23. The study also found no significant association between FGF23, calcium, phosphate, iPTH, creatinine, urea, and plaque score, and the presence of periodontitis in CKD patients. Given that this study is a pilot study, further research is warranted to explore these relationships in greater detail and to confirm these findings in a larger and more controlled research setting.
